# Dipotassium Glycyrrhizate Inhibits HMGB1-Dependent Inflammation and Ameliorates Colitis in Mice

**DOI:** 10.1371/journal.pone.0066527

**Published:** 2013-06-19

**Authors:** Roberta Vitali, Francesca Palone, Salvatore Cucchiara, Anna Negroni, Leonardo Cavone, Manuela Costanzo, Marina Aloi, Anna Dilillo, Laura Stronati

**Affiliations:** 1 Department of Radiobiology and Human Health, ENEA, Rome, Italy; 2 Department of Pediatrics and Infantile Neuropsychiatry, Pediatric Gastroenterology and Liver Unit, Sapienza University of Rome, Rome, Italy; 3 Department of Preclinical and Clinical Pharmacology, University of Florence, Florence, Italy; National Institutes of Health, United States of America

## Abstract

**Background:**

High mobility group box-1 (HMGB1) is a DNA-binding protein that is released from injured cells during inflammation. Advances in targeting HMGB1 represent a major challenge to improve the treatment of acute/chronic inflammation.

**Aim:**

This study is aimed at verifying whether the inhibition of HMGB1 through dipotassium glycyrrhizate (DPG) is a good strategy to reduce intestinal inflammation.

**Methods:**

Human colon adenocarcinoma cell line, HT29, human epithelial colorectal adenocarcinoma, Caco2, and murine macrophage cell line, RAW 264.7, were cultured to investigate the effect of DPG on the secretion of HMGB1. Acute colitis was induced in C57BL/6 mice through administration of 3% dextran sodium sulphate (DSS); a combined treatment with DSS and 3 or 8 mg/kg/day DPG was used to investigate the effects of DPG on intestinal inflammation. Animals were euthanized at seventh day and colonic samples underwent molecular and histological analyses.

**Results:**

DPG significantly reduces *in vitro* the release of HMGB1 in the extracellular matrix as well as expression levels of pro-inflammatory cytokines, TNF-alpha, IL-1beta and IL-6, by inhibiting HMGB1. Moreover, DPG significantly decreases the severity of DSS-induced colitis in mice. Murine colonic samples show decreased mRNA levels of pro-inflammatory cytokines TNF-alpha, IL-1beta and IL-6, as well as HMGB1 receptors, RAGE and TLR4. Finally, HMGB1, abundantly present in the feces of mice with DSS-induced colitis, is strongly reduced by DPG.

**Conclusions:**

HMGB1 is an early pro-inflammatory cytokine and an active protagonist of mucosal gut inflammation. DPG exerts inhibitory effects against HMGB1 activity, significantly reducing intestinal inflammation. Thus, we reason that DPG could represent an innovative tool for the management of human intestinal inflammation.

## Introduction

High mobility group box 1 (HMGB1) is a DNA-binding nuclear protein that, like other endogenous molecules termed alarmins or DAMPs (Damage Associated Molecular Patterns), can be released into the extracellular milieu during states of cellular stress or damage and subsequently activate the immune system and promote inflammation [1], [2]. To exert these activities, HMGB1 must transit from the nucleus, through the cytoplasm, to the outside of the cell. HMGB1, following a number of post-translational modifications, is actively secreted and forms highly inflammatory complexes with ssDNA, LPS, IL-1beta, and nucleosomes, which interact with TLR9, TLR4, IL-1R, and TLR2 receptors, respectively [3], [4]. These complexes elicit the release of inflammatory cytokines more effectively than each molecule alone [5], [6]. HMGB1 also induces the recruitment of inflammatory cells [7]–[9], contributes both to dendritic cell maturation [10], [11] and proliferation of activated T cells [12]. For all these reasons, HMGB1 is actually considered a potent inflammatory mediator and has been implicated in several inflammatory and auto-immune disorders, such as sepsis, rheumatoid arthritis, lupus erythematosus, myositis, diabetes and, ultimately, inflammatory bowel disease (IBD) [1], [13]–[17].

It is currently believed that advances in targeting HMGB1 represents a major challenge to improve the treatment of acute/chronic inflammation as well as infection and ischemia-reperfusion induced injury. Hence, a growing number of HMGB1 inhibitors, ranging from neutralizing antibodies, endogenous hormones, to medicinal herb-derived small molecule, has been developed [18]–[22]. Among these, glycyrrhizin, a glycoconjugated triterpene produced by the licorice plant, *Glycyrrhiza glabra*, with known anti-inflammatory properties, is thought to inhibit the cytokine activity of HMGB1 and improve inflammation during acute/chronic phases of diseases such as hepatitis, myocarditis or lung injury [23]–[27].

We previously demonstrated that HMGB1 is abundantly secreted by human inflamed intestinal tissues of pediatric patients with IBD and, once released, it behaves as a cytokine-like pro-inflammatory molecule by up-regulating other pro-inflammatory mediators. Hence this protein was supposed to play an active role in the pathogenesis of the disease. We also suggested that targeting HMGB1 may represent a major challenge to improve the treatment of acute/chronic inflammation [17].

Therefore, in the present study we sought to understand whether the inhibition of HMGB1 through glycyrrhizin, is a good strategy to reduce intestinal inflammation. We show that dipotassium glycyrrhizate (DPG), a salt of glycyrrhizin, impairs HMGB1 activity and its administration causes a decreased loss of body weight and large intestine length as well as an amelioration in clinical and histological scores in mice with DSS-induced colitis. Due to the apparent lack of adverse side-effects of DPG, we believe that these findings may open new perspectives in the setting of alternative strategies for the treatment of human intestinal inflammation.

## Materials and Methods

### Ethic Statement

Experimental procedures were previously approved by the Ministry of Health and the study was carried out in accordance with the Italian regulations on animal welfare. The protocol was approved by the Committee on the Ethics of Animal Experiments of the Italian National Agency for New Technology, Energy and Sustainable Economic Development (ENEA) (Permit Number: 131/2012-B).

### Cell Culture

The murine macrophage-like cell line, RAW264.7, was cultured in RPMI 1640 medium, the human intestinal colorectal adenocarcinoma cell line, Caco2, in Dulbecco’s modified Eagle’s medium and the human colon carcinoma cell line HT29 in McCoy’s 5A medium. All culture media contained 10% fetal calf serum (FCS), 2 mM L–glutamine, 100 U/ml penicillin and 100 µg/ml streptomycin (Biochrom, Berlin, Germany) at 37°C, 5% CO_2_. All cell lines were purchased from ATCC (Rockville, MD, USA). Cells were seeded with a cell number of 0.6 × 10^5^ per well in six multiwell plates. Inductions were performed by adding to culture medium (without serum) 1 µg/ml LPS (Sigma, St. Louis, MO), 1 or 10 µg/ml BoxB, the truncated form of the protein consisting of the B box domain, (HMGBiotech, Milan, Italy) in presence or absence of 300 µM DPG (Sigma) dissolved in PBS. Total RNA and proteins were extracted for real-time PCR (RT-PCR) and western blot analyses. Supernatants were collected after 24 and 48 h, briefly centrifuged, and extracellular HMGB1, IL-6 and TNF-alpha analysed by western blot.

### Animals

C57BL/6 female mice (8 to 9 weeks of age) were purchased from the animal housing unit of Harlan Laboratories, SRL. Animals were housed in collective cages at 22+/−1°C under a 12-hour light/dark cycle and with food and water provided ad libitum.

Induction of ColitisAcute colitis was induced through administration of dextran sodium sulphate (DSS, molecular mass, 36,000–50,000 Da, MP Biomedicals, Santa Ana, CA), dissolved in autoclaved drinking water, for 7 days. Mice, 10 for group, were randomly divided into four experimental groups: the control group received regular drinking water; DSS-treated group was given a solution with 3% (w/v) DSS; two groups received a combined treatment with 3% DSS and 3 mg/kg/day or 8 mg/kg/day DPG (DMG Italia Srl, Pomezia, italy), diluted in PBS, by oral gavage. Mice were daily checked for behavior, body weight, stool blood and consistency.

### Assessment of DSS-induced Colitis and Histological Score

All animals were daily examined and the clinical score (CS) was assessed according to the criteria of Maxwell et al. [28] by assessing stool consistency (0 for normal stool, 1 for moist/sticky stool, 2 for soft stool, 3 for diarrhea), presence of blood in stool (0 for no blood, 1 for evidence of blood in stool or around anus, and 2 for severe bleeding) and general appearance of the animal (0 was assigned if normal, 1 for ruffled fur or altered gait, 2 for lethargic or moribund). Mice were daily weighed, and the percentage of weight loss was calculated in relation to the starting weight using the formula: [(Weight on day X- Initial weight)/Initial weight] × 100 [28].

The 7^th^ day, animals were euthanized, colons removed and examined for weight, length (measured from the anus to the top of the cecum) and stool consistency. Distal colonic specimens were frozen in liquid nitrogen or fixed immediately in a 10% (w/v) formalin solution for further analyses. Stool specimens were also collected and stored at −20°C.

For histological analysis, fixed colonic tissues were embedded in paraffin, sectioned (4 µm thickness), mounted on glass slides, and deparaffinized. Slices were stained using standard hematoxylin and eosin (H&E) techniques. Samples were analyzed by light microscopy and were scored according to the criteria of Maxwell et al. [28]. Experiments were made in double-blind.

### Real-time PCR

Total RNA was isolated from cell line RAW264.7 or from mouse colonic tissues using the RNeasy kit (QiaGen GmbH, Hilden, Germany), and 1 µg of total RNA was reverse transcribed by a High-Capacity cDNA Reverse Transcription Kit (Applied Biosystems, Foster City, CA). The RT-PCR amplifications were done with an ABI PRISM 7300 Sequence Detection System using the SYBR Green kit (Applied Biosystems). Specific primers were identified using the Primer Express v.3.0 (Applied Biosystems) provided with the ABI Prism 7300 sequence detector.

The following primers were used:

HMGB1 forward (fwd) primer: 5 ′- GCCTCGCGGAGGAAAATC -3 ′; HMGB1 reverse (rvs) primer: 5′-AAGTTTGCACAAAGAATGCATATGA-3′; TNF-alpha fwd primer: 5 ′CAGACCCTCACACTCAGATCATCTT -3′; TNF-alpha rvs primer: 5′TCGTAGCAAACCACCAAGTGG -3 ′; IL-1beta fwd primer: 5 ′- CGAGGCAGTATCACTCATTG -3′; IL-1beta rvs primer: 5 ′- CGTTGCTTGGTTCTCCTTGT -3 ′; IL-6 fwd primer: 5 ′-CAAGTCGGAGGCTTAATTACACATG -3 ′; IL-6 rvs primer: 5 ′- AGAAAAGAGTTGTGCAATGGCA -3 ′; RAGE fwd primer: 5 ′-TCCCGATGGCAAAGAAACACT-3 ′; RAGE rvs primer: 5 ′-CAGCTCTGACCGCAGTGTAA-3 ′; TLR-4 fwd primer: 5 ′- CGCTTTCACCTCTGCCTTCACTACAG -3 ′;TLR-4 rvs primer: 5 ′- ACACTACCACAATAACCTTCCGGCTC -3 ′;GAPGH fwd primer 5 ′- AACTTTGGCATTGTGGAAGG -3 ′; GAPGH rvs primer 5 ′- CACATTGGGGGTAGGAACAC -3 ′.

For *in vitro* experiments the expression level of each mRNA was assessed using the standard curve method and GAPDH was used for normalization.

For *in vivo* experiments, the expression level of each mRNA was assessed using the comparative CT(^ΔΔ^C_T_) method as described by the manufacturer.

### Protein Extraction

Cell pellets or mouse colonic tissues were suspended in ice-cold lysis buffer (50 mM Tris (pH 7.4), 5 mM EDTA, 250 mM NaCl, 0.1% Triton X-100, 1 mM phenylmethylsulfonyl fluoride, 5 µg/ml aprotinin, 5 µg/ml leupeptin, and 1 mM sodium orthovanadate), homogenized and incubated in ice for 20 min. Samples were centrifuged at 14,000 r.p.m. for 10 min and supernatants collected and analyzed by western blot.

### Nuclear Cytoplasmic Separation

Nuclear and cytoplasmic protein fractionation was performed on a subset of murine colonic samples. Briefly, frozen tissue was added to 0.5 ml of fractionation buffer (10 mM HEPES (*N* -2-hydroxyethylpiperrazine- *N* -2-ethanesulfonic acid) (pH 7.4), 50 mM KCl, 15 mM MgCl_2_, 0.1 mM EGTA, 1 mM dithiothreitol, 1 mM phenylmethylsulfonylfluoride, 5 µg/ml leupeptin, and 5 µg/ml aprotinin) and homogenized. Cells were allowed to swell on ice for 30 min and the homogenate was centrifuged for 2 min at 5,000 r.p.m. The supernatant was collected and used for cytoplasmic protein analysis. The nuclear pellet was suspended in 20 µl of ice-cold nuclear extraction buffer (20 mM HEPES (pH 7.9), 350 mM NaCl, 1.5 mM MgCl_2_, 0.2 mM EDTA, 25% glycerol, 1 mM phenylmethylsulfonyl fluoride, 5 µg/ml leupeptin, and 5 µg/ml aprotinin) and incubated in ice for 20 min. Samples were freeze-thawed three times, centrifuged for 15 min at 13,000 r.p.m., supernatants were collected and used for nuclear protein analysis.

### Fecal Extraction

Murine stool specimens were collected and stored at −80°C. Each sample was resuspended in denaturing buffer (Life Technologies Ltd, Carlsbad, CA) to obtain a final concentration of 500 mg/ml of feces. Samples were vortexed for 1 min and placed in orbital shaking for 1 h at room temperature. After being centrifuged for 5 min at 10,000 r.p.m. at 4°C, supernatants (fecal extracts) were collected and the total protein concentration was determined by the Bradford assay (Bio-Rad Laboratories, Hercules, CA).

### Immunoblot Analysis

2 µg of total proteins, 5 µg of fecal extracts or 10 µl of cellular medium were fractionated by sodium dodecyl sulfate-polyacrylamide gel electrophoresis to detect HMGB1. Proteins were transferred in polyvinylidene fluoride membrane (Bio-Rad) and blocked with TBS-T (Tris-buffered saline with Tween-20) containing 5% non-fat dry milk. Anti-HMGB1 (1∶1,000; Sigma), anti-β-actin (1∶5,000; Sigma) anti-TNFalpha (1∶1,000; Abcam), anti-IL-6 (1∶500; LifeTechnologies) antibodies were diluted in TBS-T containing 3% non-fat dry milk and incubated overnight at 4°C. Membranes were washed in TBS-T, incubated for 1 h with horseradish peroxidase-conjugated secondary antibody (Santa Cruz Biotechnology Inc, Santa Cruz, CA), washed in TBS-T, and developed with ECL-Plus (GE Healthcare, Life Science, Uppsala, Sweden). Densitometrical analyses of the blots were performed using the Software ImageQuant (GE Healthcare Life Science, Uppsala, Sweden).

### Comassie Blue Staining

5 µg of fecal extracts were fractionated by SDS-polyacrilamide gel and stained by Comassie Blue as a loading control for western blot on stool sample. Briefly, after the electrophoresis, the gel was first stained in the Comassie Blue solution (Bio-Rad) for 1 hour, then destained in the destaining solution (40% methanol, 10% acetic acid).

### Immunohistochemistry

Sections (4 µm) of paraffin-embedded intestinal colonic samples from mice were prepared following the standard protocol. Briefly, sections were dewaxed for 20 min at 56°C and incubated in citrate buffer pH 6.0 for 20 min at 95°C. Afterward, sections were washed in water for 5 min and peroxidases inhibited by incubation in 3% H_2_O_2_ for 10 min. Sections were treated with 5% bovine serum albumin (Santa Cruz) for 20 min and incubated with primary anti-HMGB1 antibody (Sigma), diluted 1∶1,000 in phosphate-buffered saline for 1 h at room temperature in a moist chamber. They were then washed in phosphate-buffered saline, incubated for 30 min with the secondary anti-rabbit antibody (Dako North America, Carpinteria,CA), and washed again in phosphate-buffered saline. The DAB detection kit (DAKO) was used, as suggested by the providers, to visualize the antigen. Finally, samples were stained with H&E.

### Statistics

All experiments were repeated three times. Data were given as mean ± standard deviation (sd). All statistical analyses were carried out using GraphPad InStat software. The Kolmogorov—Smirnov test was used to assess whether data were sampled from populations following the gaussian distribution. Comparison among groups was performed using the parametric Anova ordinary test (significance taken as p<0.01). Multiple comparisons were made by using Tukey-Kramer Multiple Comparison Test.

## Results

### DPG Significantly Influences in vitro the Release of Extracellular HMGB1

**Figure 1:**
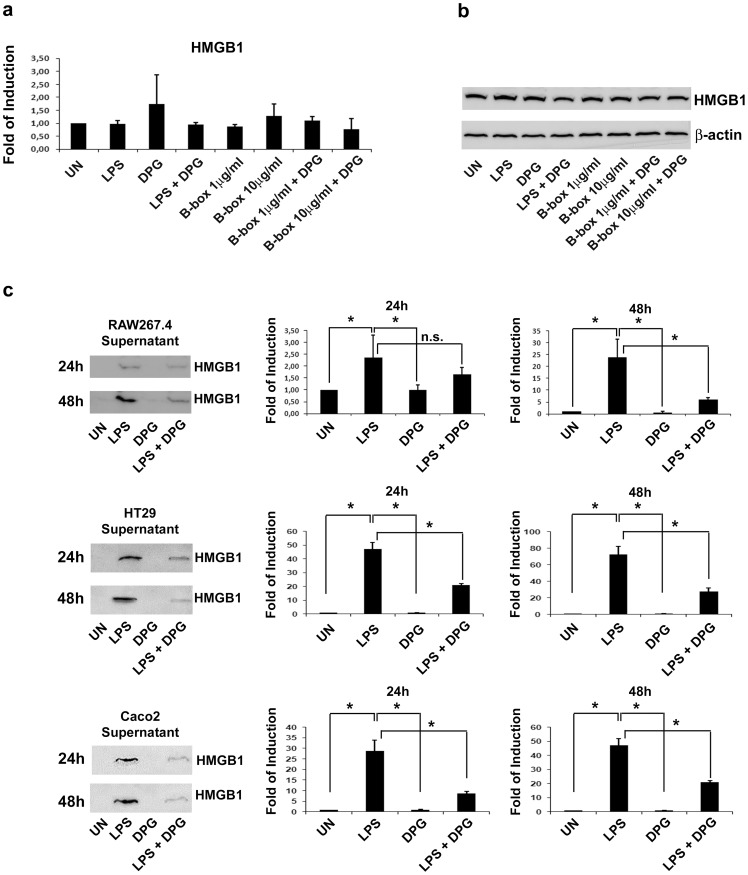
In vitro effects of DPG on gene/protein expression and extracellular level of HMGB1. RAW267.4 cell line was treated for 4 hours with LPS, DPG and B-box (**a**) Quantitative real-time PCR analysis. Data represent the target gene expression normalized to the reference gene; (**b**) western blot assay; (**c**) RAW267.4, HT29 and Caco2 cell lines were treated for 24–48 hours with LPS, DPG; extracellular HMGB1 was then analysed in the culture medium by western blot. Densitometric analysis is showed. Values (optical density units, O.D.) are the mean ± sd, of three independent experiments, referred to the optical density values of untreated samples. UN, untreated samples; LPS, lipopolysaccharide; DPG, dipotassium glycyrrhizate; B-box, recombinant truncated form of HMGB1 consisting of the B box domain. *p<0.01.

HMGB1 carries out primary functions in the nucleus, where it is abundantly expressed in almost all eukaryotic cells. It is known that, when the inflammation is triggered, HMGB1 leaves the nucleus and, through the cytoplasm, is secreted in the extracellular matrix. We performed a pilot *in vitro* experiment, by using the murine macrophage cell line, RAW 264.7, to investigate the effect of DPG on the expression of HMGB1. First we evaluated the effect of DPG on HMGB1 gene/protein expression. To trigger inflammation, cells were exposed for 4 hours to LPS or to HMGB1 B box (B-box), which is the recombinant truncated form of the full protein consisting of the pro-inflammatory component, the B box domain, (1 and 10 µg/ml), in presence or absence of DPG. As expected, gene/protein expression levels of cellular HMGB1 did not change in response to LPS nor to DPG, due to the physiological large amount of the protein inside the cell (Fig. 1a, 1b). Then, to evaluate the effect of DPG on HMGB1 secretion, we exposed cells for 24 and 48 hours to LPS, in presence or absence of DPG. As a main source of HMGB1 in vivo are epithelial cells, we performed these experiments on two other cell lines, the human intestinal colorectal adenocarcinoma cell line, Caco2, and the human colon carcinoma cell line HT29, in addition to the macrophage RAW 264.7. Results obtained were the same for all the three cell lines used. We observed a significant increase of the secreted HMGB1 in the culture medium at both exposure times (p<0.01); however, this amount was significantly reduced in presence of DPG (p<0.01) at 48 hours in RAW264.7 and at both exposure times in Caco2 and HT29, demonstrating the ability of DPG to influence the release of HMGB1 (Fig. 1c).

### DPG Reduces in vitro Expression Levels of Pro-inflammatory Cytokines, TNF-alpha, IL-1beta and IL-6, through Inhibition of HMGB1

**Figure 2:**
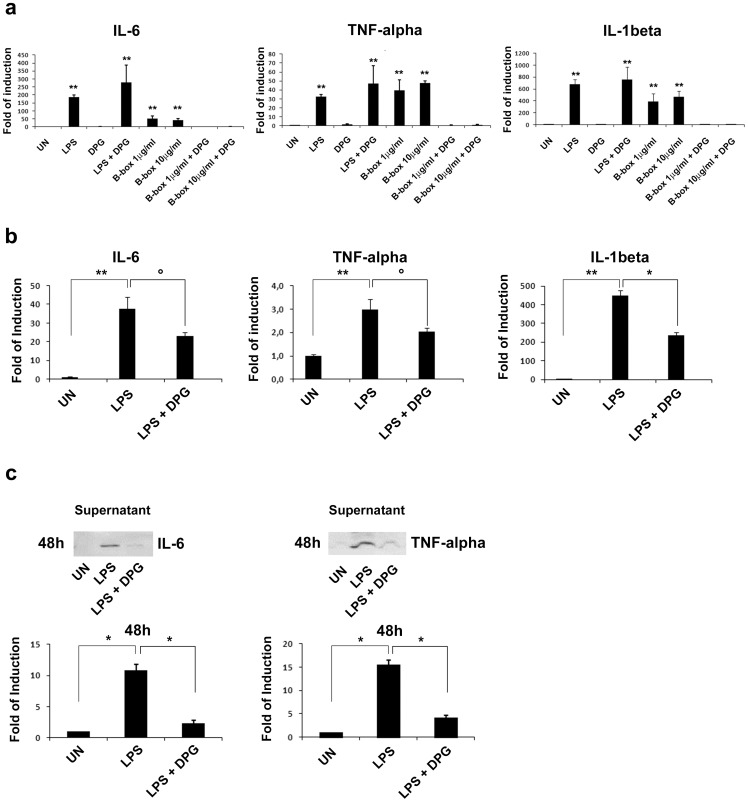
In vitro effects of DPG on gene/protein expression of pro-inflammatory cytokines, IL-6, TNF-alpha and IL-1beta. RAW267.4 cell line was treated for: (**a**) 4 hours with LPS, DPG and B-box. Cytokine mRNA expression was analysed by Quantitative real-time PCR. Data represent the target gene expression normalized to the reference gene. The error bars represent sd. (**b**) 48 hours with LPS and DPG. Cytokine mRNA expression was analysed by Quantitative real-time PCR. (**c**) 48 hours with LPS and DPG. Cytokine presence in the culture medium was analysed by western blot. Densitometric analysis is showed. Values (optical density units, O.D.) are the mean ± sd, of three independent experiments, referred to the optical density of untreated samples. UN, untreated samples; LPS, lipopolysaccharide; DPG, dipotassium glycyrrhizate; B-box, recombinant truncated form of HMGB1 consisting of the B box domain. °p<0.05; p<*<0.01;**p<0.001.

In order to understand whether DPG is able to influence the production of pro-inflammatory cytokines by inhibiting HMGB1 activity and, thus, to modulate inflammation, we treated for 4 hours RAW 264.7 cells with B-box (1 and 10 µg/ml) or LPS, in presence or absence of DPG and analysed gene expression of pro-inflammatory cytokines, TNFalpha, IL-1beta and IL-6. mRNA expression of all cytokines increased after both inflammatory stimuli, but, only when inflammation was B box-induced, cells treated with DPG exhibited a dramatic decrease of cytokine mRNA expression (p<0.001), suggesting that DPG is able to down-regulate inflammation only when it is HMGB1-mediated (Fig. 2a). Besides, to investigate the effect of DPG at longer times, we exposed cells to LPS and analysed cytokine expression as well as production, by real time PCR and western blot on culture medium, after 24 and 48 hours. We observed a significant reduction of both cytokine expression and production (p<0.05; p<0.01) at 48 (Fig. 2b, 2c), but not 24 hours (data not shown).

### DPG Significantly Decreases the Severity of Acute DSS-induced Colitis in Mice

**Figure 3:**
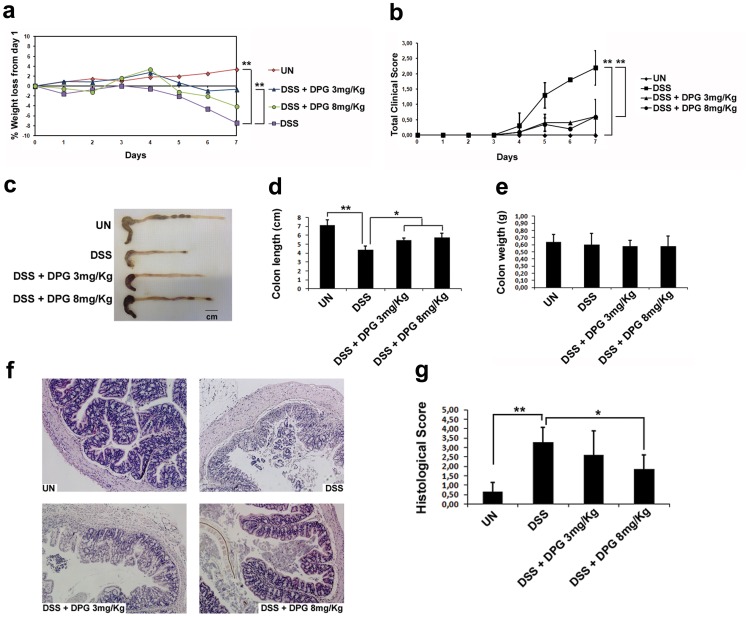
DPG reduces DSS-induced colitis in mice. DSS-treated C57BL/6 mice were administered with two different doses (3 and 8 mg/Kg) of DPG for 7 days. Following parameters were analysed: (**a**) weight loss; (**b**) total clinical score (stool consistency, presence of blood in stool and general appearance) (**c, d**) colon length (**e**) colon weight (**f**) morphology of the colon evaluated through histological analysis (40xmagnification in the image) (**g**) histological score. Values are mean ± sd of three independent experiments. UN, untreated animals; DSS, dextran sulfate sodium; DPG, dipotassium glycyrrhizate. *p<0.01; **p<0.001.

C57BL/6 mice were divided into four experimental groups (10 animals for each group): one group was fed only with water and used as negative control; one group was administered with DSS for 7 days to induce colitis and used as positive control; the other two groups were administered with DPG, parallely to DSS, at the dose of 3 or 8 mg/Kg, respectively. The following parameters were used to estimate changes at the 7^th^ day after DPG and DSS administration: body weight, clinical score (resulting from stool consistency, presence of blood in stools and general mouse appearance scores), histological score and large intestine length/weight. Results showed that mice exposed to DPG had a decreased loss of the body weight (p<0.001) at both doses, but after all at the dose of 3 mg/Kg (60% decreased weight loss) as compared to positive control as well as an evident amelioration of the total clinical score (p<0.001) (Fig. 3a, 3b). Moreover, DPG was shown to reduce the loss of length (p<0.01), but not of weight, of the large intestine as compared to mice with DSS-induced colitis (Fig. 3c, 3d, 3e). Anti-inflammatory effects of DPG was further confirmed by the histological analysis which showed an important recovery of the morphological features of the intestinal mucosa and a significant reduction of the histological score (p<0.01) after DPG treatment (Fig. 3f, 3g).

### HMGB1 Level is Strongly Increased in the Cytoplasm of Murine Inflamed Cells but it Dramatically Decreases after DPG Treatment

**Figure 4:**
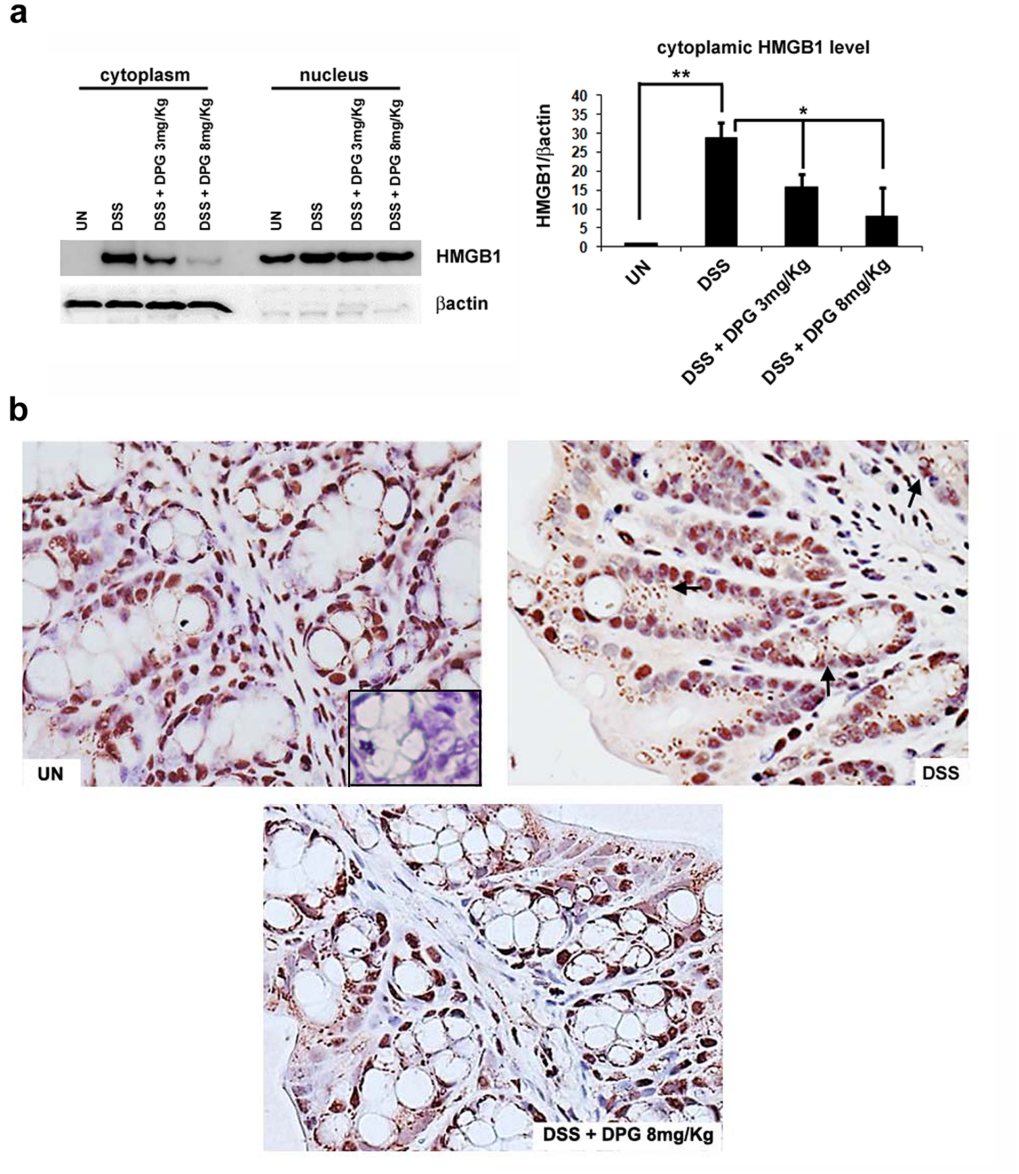
DPG reduces HMGB1 translocation from the nucleus to the cytoplasm in murine inflamed colonic tissues. Nuclear and cytoplasmic protein fractionation was performed on a subset of murine colonic samples; (**a**) HMGB1 protein expression assessed by Western blot. Anti-β actin was used as cytoplasmic protein control. Densitometric analysis is showed. Values are the mean ± sd of three independent experiments. (**b**) HMGB1 intracellular localization was evaluated through histochemical staining. Staining control is showed in the box. Arrows indicate secreted HMGB1. UN, untreated animals; DSS, dextran sulfate sodium; DPG, dipotassium glycyrrhizate. *p<0.01; **p<0.001.

HMGB1 is a nuclear protein actively shifted from the nucleus to the extracellular matrix, through the cytoplasm, upon inflammatory stimuli. To investigate the effect of DPG on HMGB1 release, nuclear and cytoplasmic HMGB1 fractions, extracted from colonic bioptic specimens of mice with DSS-induced colitis and mice treated with DSS and DPG, were separately analysed. The expression analysis showed that cytoplasmic HMGB1 was much higher in inflamed tissues as compared to uninflamed ones, while nuclear protein, as expected, was unchanged. However, the use of DPG significantly reduced cytoplasmic levels of HMGB1. The increase of cytoplasmic HMGB1 in inflamed tissues and its reduction after DPG treatment were also confirmed by histochemical analysis (Fig. 4).

### DPG Treated Mice Show Significantly Decreased mRNA Levels of Pro-inflammatory Cytokines TNF-alpha, IL-1beta and IL-6, as well as HMGB1 Receptors RAGE and TLR4

**Figure 5:**
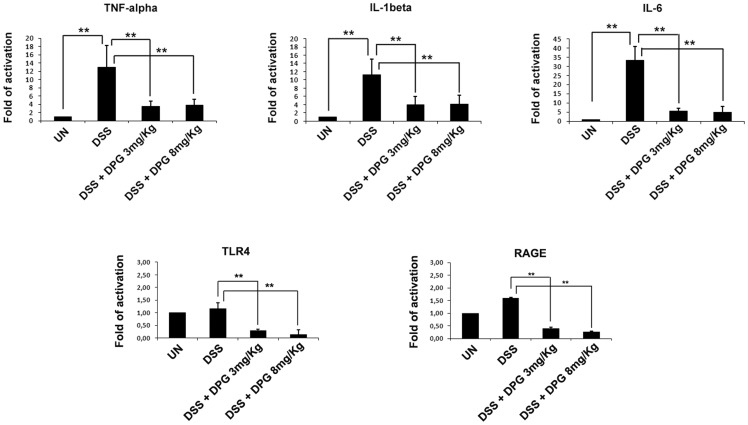
DPG reduces mRNA expression of pro-inflammatory cytokines, TNF-alpha, IL-1beta and IL-6, as well as HMGB1 receptors, TLR4 and RAGE, in inflamed tissues of DSS-treated mice. Quantitative real-time PCR. Data represent the target gene expression normalized to the reference gene. Values are mean ± sd of three independent experiments. UN, untreated animals; DSS, dextran sulfate sodium; DPG, dipotassium glycyrrhizate. **p<0.001.

Tissue samples taken from the large colon of mice with DSS-induced colitis and mice treated with DSS and DPG were analysed to assess the expression levels of pro-inflammatory cytokines, TNF-alpha, IL-1beta and IL-6, and confirm previous *in vitro* results about the ability of DPG to break down the inflammatory response. Results showed a dramatic decrease of all cytokine mRNA levels in mice treated with DPG as compared to DSS-treated mice (p<0.001) (Fig. 5). Besides, as it has been suggested that HMGB1 itself can signal through several inflammatory receptors, principally RAGE (Receptor for Advanced Glycation End products) and TLR4 (Toll-Like Receptor 4), transcripts for these receptors were also analysed. Results showed a significant decrease of RAGE and TLR4 mRNA levels in mice treated with DPG as compared to DSS-treated mice (p<0.001) (Fig. 5).

### HMGB1, Abundantly Present in the Feces of Mice with DSS-induced Colitis, is Strongly Reduced by DPG

**Figure 6:**
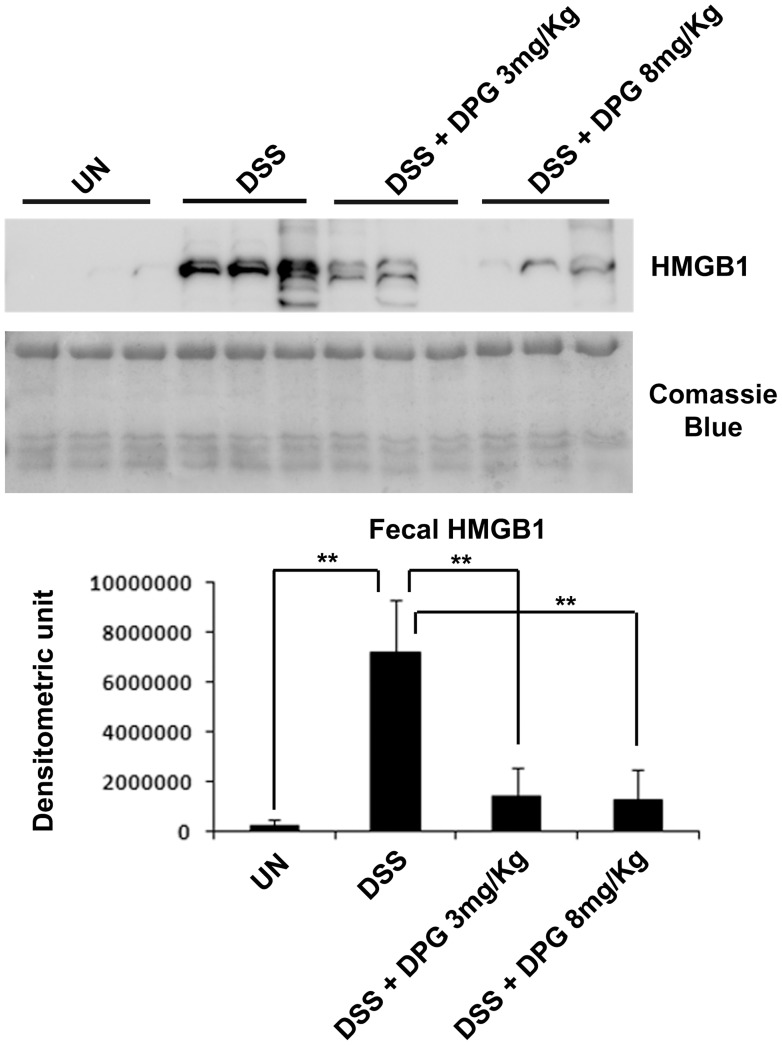
Fecal HMGB1 is strongly reduced by DPG in mice with DSS-induced colitis. Western blot of HMGB1 in stool specimens of mice. Comassie Blue staining has been used as loading control. Densitometric analysis is showed. Values are the mean ± sd of three independent experiments. UN, untreated animals; DSS, dextran sulfate sodium; DPG, dipotassium glycyrrhizate. **p<0.001.

In a previous paper, we demonstrated that HMGB1 protein expression is significantly increased in the stools of IBD patients as compared to controls [17]. In the present study we investigated the presence of HMGB1 in the stools of control mice or mice with a DSS-induced colitis and found that, like in humans, the protein is strongly expressed in mice with colitis (p<0.001), but not in controls. Furthermore, mice treated with DPG showed a significant (p<0.001) reduction of HMGB1 protein expression levels (Fig. 6).

## Discussion

HMGB1 plays a critical role at the intersection of the host inflammatory response to sterile and infectious threat and targeting HMGB1 is currently considered a very interesting novel strategy for the treatment of inflammatory diseases.

Some studies have recently explored the ability of glycyrrhizin to attenuate inflammation by suppressing or reducing HMGB1 activity. Only two groups, to our knowledge, have investigated, in rat models, the use of glycyrrhizin for the management of the intestinal inflammation reporting encouraging results. However, both papers do not correlate the effect of glycyrrhizin to HMGB1 inhibition [29], [30].

In our study, we aimed at assessing the usefulness of glycyrrhizin for the treatment of murine experimentally-induced colitis by examining a wider range of molecular and morphological end-points and highlighting the close relation between glycyrrhizin efficacy and HMGB1 reduced activity. For these purposes, we first carried out a pilot *in vitro* study to understand whether glycyrrhizin, used in its salt form, the DPG, is able to reduce the secretion of HMGB1 in two epithelial cell lines, Caco2 and HT29, and in the macrophage RAW 264.7 cell line treated with LPS and cultured for 24–48 hours. Results showed that DPG significantly decreases the release of the extracellular HMGB1 starting at 24 hours in Caco2 and HT29 and at 48 hours in RAW264.7, as evaluated by measuring the protein amount in cell culture supernatants.

Furthermore, we investigated the effect of DPG on the inflammation specifically induced by HMGB1. Thus, we stimulated RAW 264.7 cells for 4 hours with LPS or alternatively, with HMGB1 B-Box, the segment of the protein showing inflammatory properties, and then analysed mRNA expression of the known inflammatory mediators, TNF-alpha, IL-1beta and IL-6. We found that cytokine gene expression increases after both inflammatory triggers, but DPG reduces cytokine induction only after HMGB1 B-Box exposure. As HMGB1 is physiologically released out of the cells after 16–24 hours, we also investigated the effect of DPG both on the expression of cytokines that on their presence in the culture medium in LPS-treated cells for 24 and 48 hours. We found that cytokine expression is significantly decreased at 48, but not 24 hours. This result is in good agreement with that previously obtained which showed that DPG significantly inhibits HMGB1 secretion at 48 hours, while the effect is much lower at 24 hours.

In conclusion, we show that DPG is a good down-regulator of HMGB1-triggered inflammation. Given the very low cellular permeability of DPG, we reason that its anti-inflammatory effects should be ascribed to the drug’s ability to bind and inactivate extracellular HMGB1 [31], thereby halting the well-known vicious cycles of pro-inflammatory signals triggered by extracellular HMGB1 itself. In keeping with this, Mollica et al. recently reported that DPG directly binds HMGB1 boxes, without distorting their secondary structure, and so explaining, at least in part, the molecular mechanisms underlying the anti-inflammatory properties of DPG [31].

More interestingly, we investigated the DPG anti-inflammatory effect in a cohort of C57BL/6 mice treated with DSS to induce colitis. Our results demonstrate that administration of DPG causes a remarkable reduction of acute colitis, in terms of decreased loss of body weight and large intestine length as well as recovery of clinical and histological scores. Besides, mRNA expression of TNF-alpha, IL-1beta and IL-6 were significantly down-regulated in the inflamed tissues of DPG+DSS-treated mice as compared to DSS-treated mice. Hence, *in vivo* results confirm that DPG potently reduces inflammation.

In addition, as it is known that HMGB1 may activate TLR4 and RAGE receptors, creating a functional tripod that contributes to up-regulate pro-inflammatory mediators, gene expression analysis of both receptors was also made in the same murine samples; results showed a clear decrease in their mRNA levels after DPG treatment. The evidence that HMGB1 receptors are strongly decreased further suggests that DPG activity is based on a HMGB1 inhibitory mechanism. To strengthen this interpretation, we examined the effect of DPG on the nuclear and cytoplasmic cellular fractions of HMGB1, separately. Indeed, it is commonly accepted the mechanism for which HMGB1, although predominantly located in the nucleus, where it is largely present displaying a DNA-bending activity, may translocate, through the cytoplasm, to the extracellular space, after an inflammatory trigger and assume the pro-inflammatory behavior [1]. Therefore, nuclear and cytoplasmic fractions were extracted from murine colonic tissues and individually analysed for HMGB1. We show that the protein is increased in the cytoplasm, but not in the nucleus, of murine inflamed cells and the DPG treatment is able to decrease only the cytoplasmic HMGB1 level. In conclusion, we propose the model whereby glycyrrhizin, by targeting HMGB1, severely impairs the positive autoregulatory loop that is established between the secreted pro-inflammatory HMGB1 and the other inflammatory mediators that it can upregulate, itself included. The break of this loop progressively reduces the inflammation and allows the tissue recovery.

Finally, we observed that HMGB1 is present in a significant amount in the stools of mice with colitis as compared to controls, where it is almost undetectable. These results confirms those previously obtained in the stools of IBD patients, where the presence of the protein directly correlated to the grade of mucosal inflammation and therefore it was proposed as a valuable novel biomarker of intestinal inflammation 17. Moreover, we show, in the present study, that murine fecal HMGB1 is strongly decreased by DPG treatment, still supporting the role of the latter as a down-regulator of inflammation.

### Conclusions

Present data evidence that secreted HMGB1 is a pro-inflammatory cytokine and an active protagonist of gut mucosal inflammation. Several methods have been proposed for inhibiting HMGB1 secretion and ensuing inflammation. In this study, we explored *in vitro* and *in vivo* the potential of DPG, a HMGB1 inhibitor, as an anti-inflammatory compound and showed that it strongly ameliorates DSS-induced colitis in mice. We reason that DPG may represent an innovative tool for the management of human intestinal inflammation.
